# The Few Who Made It: Commercially and Clinically Successful Innovative Bone Grafts

**DOI:** 10.3389/fbioe.2020.00952

**Published:** 2020-09-01

**Authors:** Ignacio Sallent, Héctor Capella-Monsonís, Philip Procter, Ilia Y. Bozo, Roman V. Deev, Dimitri Zubov, Roman Vasyliev, Giuseppe Perale, Gianni Pertici, Justin Baker, Peter Gingras, Yves Bayon, Dimitrios I. Zeugolis

**Affiliations:** ^1^Regenerative, Modular & Developmental Engineering Laboratory, National University of Ireland Galway, Galway, Ireland; ^2^Science Foundation Ireland Centre for Research in Medical Devices (CÚRAM), National University of Ireland Galway, Galway, Ireland; ^3^Division of Applied Materials Science, Department of Engineering Sciences, Uppsala University, Uppsala, Sweden; ^4^GPBio Ltd., Shannon, Ireland; ^5^Histograft LLC, Moscow, Russia; ^6^Federal Medical Biophysical Center of FMBA of Russia, Moscow, Russia; ^7^I.I. Mechnikov North-Western State Medical University, Saint Petersburg, Russia; ^8^State Institute of Genetic & Regenerative Medicine NAMSU, Kyiv, Ukraine; ^9^Medical Company ilaya^®^, Kyiv, Ukraine; ^10^Industrie Biomediche Insubri, Lugano, Switzerland; ^11^Viscus Biologics LLC, Cleveland, OH, United States; ^12^Sofradim Production, A Medtronic Company, Trévoux, France

**Keywords:** bone grafting, bioadhesives, bioactive scaffolds, cell therapies, clinical trials, commercialization

## Abstract

Bone reconstruction techniques are mainly based on the use of tissue grafts and artificial scaffolds. The former presents well-known limitations, such as restricted graft availability and donor site morbidity, while the latter commonly results in poor graft integration and fixation in the bone, which leads to the unbalanced distribution of loads, impaired bone formation, increased pain perception, and risk of fracture, ultimately leading to recurrent surgeries. In the past decade, research efforts have been focused on the development of innovative bone substitutes that not only provide immediate mechanical support, but also ensure appropriate graft anchoring by, for example, promoting *de novo* bone tissue formation. From the countless studies that aimed in this direction, only few have made the big jump from the benchtop to the bedside, whilst most have perished along the challenging path of clinical translation. Herein, we describe some clinically successful cases of bone device development, including biological glues, stem cell-seeded scaffolds, and gene-functionalized bone substitutes. We also discuss the ventures that these technologies went through, the hindrances they faced and the common grounds among them, which might have been key for their success. The ultimate objective of this perspective article is to highlight the important aspects of the clinical translation of an innovative idea in the field of bone grafting, with the aim of commercially and clinically informing new research approaches in the sector.

## Introduction

Bone’s extraordinary healing capacity can be challenged by complex fractures (i.e., injuries above critical size) or health conditions (i.e., diabetes, genetic factors, poor lifestyle), resulting in non-union fractures that can lead to long-term disability and pain (Keating et al., 2005). Bone grafting is one of the most frequently used procedures in traumatology, orthopedics, oral and maxillofacial surgery, intending to form new bone tissue at the target area (e.g., skeletal defect, atrophy region, a space between bones to be fused). Annually, half a million patients require bone repair intervention in the US and Europe (Amini et al., 2012). The global annual expenditure in bone fractures and orthobiologics is estimated at US$ 5.5 billion and US$ 4.7 billion, respectively, whilst the total cost of bone repair-related expenditure is estimated at US$ 17 billion per year (Ho-Shui-Ling et al., 2018).

Bone repair interventions are based on autografts, allografts, xenografts, and artificial scaffolds. Autografts are considered the gold standard due to their osteoinductive and osteoconductive properties, but entail important drawbacks, such as limited availability and donor-site morbidity (Fillingham and Jacobs, 2016; Morris et al., 2018; Haugen et al., 2019). Allografts and xenografts, although efficiently overcoming the aforementioned limitations, are prone to trigger immune rejection, disease transmission and their osteoinductive potential is frequently impaired due to disruptive processing (Fillingham and Jacobs, 2016; Klifto et al., 2018; Haugen et al., 2019). Alternative approaches are based in the use of artificial scaffolds specifically designed to maintain physical integrity and promote bone ingrowth at the defect site. Artificial scaffolds, also denominated bone graft substitutes, can be divided in three groups: natural or synthetic scaffolds alone, scaffolds combined with bioactive molecules and cell-based combination products (Ho-Shui-Ling et al., 2018).

Although a huge range of bone grafts and substitutes is available for clinical use, the problem of effective reconstructive treatment remains extremely challenging. Further, despite the extensive pre-clinical investigations, the translational pathway for novel technologies is slow and commonly results in minor improvements of established clinical treatments. The major impediments reside in scalability, high economic requirements and safety concerns that some of these new therapies entail (Hollister and Murphy, 2011; Bara et al., 2016).

Herein, we discuss some of the therapies that have successfully reached the clinic, serving as models in clinical translation of bone tissue engineering. This manuscript covers the use of glues, cements, tissue grafts, biohybrid scaffolds, bioactive matrices, and stem cell-loaded constructs in reported clinical trials or in well-settled clinical practices.

## From Screws to Bone Cement and Glue

The implantation of plates and screws to fix bone fractures is a common practice in orthopedic surgery since the beginning of the 20th century. These implants have evolved substantially with the development of new materials, designs, and clinical implantation strategies (Augat and von Rüden, 2018). The primary function of plates and screw implants is to provide mechanical stability to bone fracture fragments. In non-locked plates as the screws are tightened and go into tension, the resulting friction between the plate and the bone stabilizes the fracture and results in a load-sharing device (Egol et al., 2004). The Gamma Locking Nail^®^ (Stryker) is an example of an intramedullary fixation system whose design has been shown effectiveness in providing mechanical stability as an intramedullary fixation device in hundreds of thousands implantations to date (Queally et al., 2014). However, implant complications still occur at considerable rates (up to 20%) and include screw cut-out through poor bone quality, re-fractures and infections that necessitate revision surgeries (Ahrengart et al., 2002; Schliemann et al., 2015; Ma et al., 2017). In such complications, bone quality plays a vital role and osteoporotic or low-density bone leads inevitably to higher risks of implant failure. Other factors, such as implant position and anatomical fracture reduction, influence this complication rate independently of the screw design (Mueller et al., 2013). Therefore, for several decades, it has been accepted that there is a clinical need for augmentation systems, which will improve the performance of the current fixation devices, in terms of osteointegration and biomechanical support, particularly in osteoporotic bone.

To satisfy this unmet clinical need, bone cements have been further developed to increase the area of contact between the screws and the bone, providing better anchoring and mechanical support (DeKeyser et al., 2019). Such bone cements have proved their clinical value in fractured osteoporotic bones (Moroni et al., 2006; DeKeyser et al., 2019; McCoy et al., 2019). This is the case in HydroSet^TM^ (Stryker), a cement employed in clinics to augment screws in cancellous bone (approved in the EU only) in both *ex vivo* (Kainz et al., 2014; Ruddy et al., 2018) and *in vivo* (Larsson et al., 2012) studies. This and other calcium cement-based products, such as BoneSave^TM^ (Stryker), BoneSource^®^ (Stryker) and Norian SRS^®^ (Synthes), have built their path into the clinic and have improved the mechanical stability of screws and outcomes in the treatment of poor quality bone (Van der Stok et al., 2011). However, limitations exist and there is still room for improvement (Van der Stok et al., 2011; DeKeyser et al., 2019; McCoy et al., 2019). Specifically, there is a major clinical need in bone surgery to attach the implant to bone and/or bone to bone, whilst improving biomechanical properties and promoting *de novo* osteoinduction (Sánchez-Fernández et al., 2019). However, there is a gap in the clinical translation of an adhesive biomaterial to meet this specific need. Surgical adhesives, such as cyanoacrylates, have been investigated and shown good mechanical properties *in vitro* (Kandalam et al., 2013), but they lack the osteoinductive potential and their degradation products induce local and systemic toxicity (Hochuli-Vieira et al., 2017; Sánchez-Fernández et al., 2019). Other adhesives, such as fibrin-based glues, present poor mechanical properties (Noori et al., 2017; Sánchez-Fernández et al., 2019). To fill this unmet need, functionalized bone cements have been developed, such as the OsStic^TM^ (GPBio), which is a bioceramic glue composed of tricalcium phosphate combined with phosphoserine, an amino acid. The amino acid triggers rapid (minutes) bonding, providing a strong fixation between tissues and biomaterials (Pujari-Palmer et al., 2018). This occurs through a hierarchical organization of an organic/inorganic interphase, where phosphoserine acts as a nucleation initiator, forming a fibrillar network and allowing the aggregation of calcium phosphate. This bone adhesive technology has already proved its safety and effectiveness in initial pre-clinical *in vivo* tests (Procter et al., 2019) and seems to have a clear pathway to clinical translation, considering that it combines a clinically used material (calcium in bone cements) and an amino acid, whose mechanism of action has been interpreted (Pujari-Palmer et al., 2018). Should further *in vivo* studies confirm the initial positive results, this is an assured pathway (combining materials with successful clinical history) to effective clinical translation to address a significant unmet clinical need.

## Tissue Grafts as Bone Graft Augmentation Devices in Dentistry – Commercial Development Challenges

Bone grafting is an extensive practice in dentistry with an increasing trend, where implant failure due to the poor fixation or loosening of the implanted grafts is a common complication (Liaw et al., 2015; Kang et al., 2019). To reduce these complications, containment materials are employed to increase the contact interface with the graft and to facilitate cellular in-growth and targeted high quality bone formation, which results in a better fixation and stabilization of the bone graft material (Larsson et al., 2016). Initially, non-resorbable synthetic polymers, such as polytetrafluoroethylene, were employed with this goal, however, they require a second surgical intervention for removal, which unavoidably increases patients’ distress and expenditure. This encouraged the employment of decellularised tissue scaffolds (Elgali et al., 2017). The significant advantages of decellularised scaffolds include high cytocompatibility and remodeling potential, which promote osteointegration and regeneration of surrounding soft tissue (Vignoletti et al., 2014; Elgali et al., 2017). Tissue grafts employed in this clinical scenario include allografts (e.g., decellularised pericardia and skin) (Adibrad et al., 2009) or xenografts (e.g., processed porcine and bovine dermis) (Wessing et al., 2017; Arunjaroensuk et al., 2018), which are extensively and successfully employed in other fields, including wound healing or hernia repair (Slater et al., 2013; Brett, 2015; Chen and Liu, 2016). Examples of these products include Creos^TM^ Xenoprotect (Nobel Biocare^TM^) or BioGuide^®^ (Geistlich), which have proved to promote bone gain and implant support in 46 patients undergoing dental surgery (Wessing et al., 2017).

For the clinical translation of these products in dentistry, special attention must be paid to their source and processing. The raw material (i.e., the tissue graft) requires extensive screening to reduce the risk of disease transmission in both allografts and xenografts, as regulated by FDA in the recognized standard ASTM F2212-11 or CE with EU Regulation 722/2012 and ISO 22442-2015, still valid also under the new European Medical Device Regulation EU 2017/745. In addition, processing of these materials (debriding, decellularization, crosslinking, etc.) must be carried out under strict cGMP or ISO standards to ensure safety, reproducibility, and scalability of the process. Examples of these standards of processing and source control include the 2014 FDA Guidance: Medical Devices Containing Materials Derived from Animal Sources (except for *in vitro* diagnostic devices), FDA’s Quality System Regulations 21 CFR 820 and the Quality Management System standard for medical devices ISO 13485-2016. Another point of stress is the sterilization of these products, which must ensure concurrently maximum safety and minimum risk of infection upon implantation (Delgado et al., 2014), in an already susceptible to infection location, the human mouth. All this processing steps must be accompanied with the preservation of the structure and composition of the graft (Delgado et al., 2015; Liaw et al., 2015); after all, these properties rationalize their use and offer them a competitive advantage over synthetic materials. Should these commercial development requirements be met, tissue grafts would have their niche ensured in the clinical translation of dentistry as augmentation systems.

## Biohybrid Grafts – Nature-Inspired Bone Substitutes

Advances in bone tissue engineering have resulted in a constant decline in the use of autografts, the gold standard in clinical practice (Miller and Chiodo, 2016; Zorica et al., 2016; Klifto et al., 2018; Haugen et al., 2019), and a parallel increase in artificial scaffolds (Morris et al., 2018; Haugen et al., 2019; Stark et al., 2019). However, the new products are far from optimal as low fusion rates and adverse effects have been reported (Zorica et al., 2016; Haugen et al., 2019). To overcome these limitations, nature-inspired bio-hybrid bone grafts (e.g., calcium-phosphate/poly-ε-caprolactone particles (Neufurth et al., 2017), silicon carbide/collagen scaffolds (BioSiC) (Filardo et al., 2014), poly(*N*-acryloyl 2-glycine)/methacrylated gelatin hydrogels (Gao et al., 2019) have been developed. These materials combine the mechanical properties of tailored synthetic polymers and the bioactive element of natural polymers or minerals. A successful example in the clinical translation is a bovine-derived mineral matrix reinforced with resorbable poly(lactic-co-caprolactone) block copolymer embedding RGD-exposing collagen fragments onto its surface (SmartBone^®^, IBI) (Pertici et al., 2014). Its design follows the “safety by design” paradigm, that is now considered one of the pillars of the new European Medical Devices Regulation. This means that each single component used is sourced in its medical-grade supply form, and its role in the overall mechanism of action is well established and supported by clinical evidences. However, such design must be accompanied by an extensive characterization (e.g., composition, microstructure, mechanical performance, cytocompatibility, preclinical model assessment) to show the safety and efficacy of the device according to international standards (e.g., ISO and ASTM). Also, production under an ISO13485:2016 standard is required to reach the clinic and market scalability. More relevantly, under the new Medical Devices Regulation, and also having in mind that the very ultimate goal is to improve patients’ health, clinical performance of innovative products, without existing equivalent products in the market, has to be evaluated during the pre-market approval process (Haugen et al., 2019).

In the case of SmartBone^®^, such a path resulted in a fully positively characterized material *in vitro* (Pertici et al., 2014, 2015), *in vivo* (Pertici et al., 2015) and in clinical trials (Abuelnaga et al., 2018; Ferracini et al., 2019), ultimately granting device certification (i.e., CE marking), which was complemented with a post-marketing surveillance in its clinical applications related to bone regeneration in various skeletal disorders. Many other bio-hybrid composites are following the same path with positive results *in vitro* and *in vivo* (Ceccarelli et al., 2017) and in preliminary clinical trials, like in the case of hydroxyapatite/collagen scaffolds (Kon et al., 2011). However, the number of successful bone substitutes in clinical translation remains very low, considering the number of research studies (e.g., 9,313 papers appear in PubMed; terms searched: “bone” and “scaffold” in Title/Abstract). The key to survive the “med-tech valley of death” is in an evidence-based approach from start to finish. This spans from identifying and understanding the unmet clinical need through to measurable clinical outcomes that prove the market differentiation value of the biohybrid medical device to both patient and payer.

## Enhancing Bone Regeneration With Bioactive Materials

Among bone autografts and substitutes, “bioactivated materials” with growth factors are considered as combination products or drugs in FDA and EMA terms, respectively. Alternative to growth factors have been developed with formulations containing cells or gene constructs that are capable of stimulating reparative osteogenesis with different regulatory status, for example, falling into the category of Advanced Therapy Medicinal Products (ATMPs) in Europe (Ho-Shui-Ling et al., 2018).

Bone regeneration is a multi-step process spatiotemporally coordinated by an array of growth factor signaling pathways (De Witte et al., 2018). Bone morphogenetic proteins (BMPs) were the first growth factors to be identified as osteoconductive and osteoinductive, in other words, being able to differentiate stem cells toward osteoprogenitor cells and promote scaffold bone tissue ingrowth (Evans, 2013). Since their FDA approval in the early 2000s, BMP-2 and BMP-7 remain the most commonly used growth factors for bone graft functionalization and constitute the active molecules of two major devices: Infuse^®^ (Medtronic) and Osigraft^®^ (Olympus), respectively (Evans, 2013). These two collagen-based bone grafts have repeatedly shown to promote bone ingrowth in FDA approved clinical spine and tibia trauma indications (Friedlaender et al., 2001; Govender et al., 2002). However, Osigraft^®^ production was discontinued and the off-label use of Infuse^®^ has resulted in reported complications (Simmonds et al., 2013; Vukicevic et al., 2014). The BMP solution component must exclusively be used with a legally approved carrier/scaffold component and for the legally approved indication. This highlights the importance of a new or improved technology that will allow for a controlled release of the bioactive cargo, to support the regulatory approval of extended clinical uses (Geiger et al., 2003; Chatzinikolaidou et al., 2010; Carragee et al., 2011).

Despite promising results in research for “smart” formulations to support improved control over growth factor release to bone regeneration sites, the reality is that most of these ambitious materials never go beyond the animal study stage (Bessa et al., 2010; De la Riva et al., 2010; Webber et al., 2015). Indeed, such products have to compete with autografts and demineralized bone matrix (DBM), in terms of bone repair or fusion efficacy, particularly when they are processed to maintain the osteoinductive and osteoconductive properties of the native bone matrix (Miron and Zhang, 2012). Most of the activated devices that made it to the clinics in the past decade are based in allografts or collagen/tricalcium phosphate scaffolds. This is the case of OsteoAMP^®^ (Bioventus Surgical) and Augment^®^ bone graft (Wright Medical Group), the former made of an allogeneic-derived matrix, especially treated to preserve a cocktail of native growth factors and the latter composed of a collagen/tricalcium phosphate composite combined with platelet-derived growth factor (PDGF-BB). Both devices promoted bone ingrowth without the need of autograft harvesting in two clinical trials with patients undergoing transforaminal lumbar or lateral interbody fusion and ankle or hindfoot arthrodesis, respectively (DiGiovanni et al., 2013; Roh et al., 2013; Krell and DiGiovanni, 2016).

The short-lived activity of growth factors in medical devices may be a concern for optimal clinical efficacy (Jabbarzadeh et al., 2008). A sophisticated new approach able to circumvent this limitation is based on the exogenous delivery of plasmid DNAs from gene-activated matrices to the host cells in the site of bone defects, to induce the endogenous production of reparative growth factors. In a recent clinical trial, a combination product based on a collagen-hydroxyapatite medical device and a plasmid DNA encoding for vascular endothelial growth factor-A (VEGF-A), showed to promote bone ingrowth in maxillofacial bone defects without causing adverse effects (Bozo et al., 2016). Similarly, Histograft (Russia) has developed an octacalcium phosphate scaffold carrying VEGF-A coding plasmid that has completed a clinical trial (NCT03076138).

Taken together, devices with bioactive molecules have proven to match or increment the regenerative capabilities of traditional bone grafts. However, the flow of technology from benchtop to clinic appears to be slow, since any new bioactive candidate should strictly comply with regulatory requirements, notably in terms of safety and efficacy (Vukicevic et al., 2014). The 510(k) process of FDA allows devices characterized as “substantially equivalent” to an existing approved device to enter fast into the market. However, bioactive scaffolds with poorly defined degradation products require significant effort to establish safety, substantially increasing time and cost associated with preclinical and clinical assessment (Webber et al., 2015; Ho-Shui-Ling et al., 2018). The design of a bioactive molecule(s) delivery system that can serve as a scaffold for cell attachment and matrix deposition, whilst promoting the active migration of cells, angiogenesis, and osteogenic cell differentiation, all in the right time and amount, appears as a mission impossible, unless more elegant, yet regulatory compliant systems, are developed (Abbah et al., 2015; Thomas et al., 2016). It is also time to realize that a single molecule approach is unlikely to result in functional repair and regeneration (Pugliese et al., 2018). However, regulatory approval of multi-cargo delivery systems is rather onerous, which encourages the use of cell therapies, considering that cells can act as a factory of trophic/bioactive molecules at the site of implantation.

## Biomaterial and Stem Cells Combining Technologies

Cell-based strategies for bone tissue engineering have a long trajectory in the research stage but have minimally contributed to current clinical practices (Mishra et al., 2016). Indeed, the introduction of cells as a component in tissue engineering entails important economic and safety concerns; the former is related to the logistics, technology and human resources necessary and the latter is related to possible immunogenicity, teratoma formation and disease transmission risks (Webber et al., 2015). As a result of the second, only those therapies that involve minimal *ex vivo* manipulation of autologous cells are FDA approved, whilst those that follow the traditional tissue engineering paradigm (*in vitro* expansion of autologous/allogeneic cells and *ex vivo* development of a cell-based construct), present a more tortuous regulatory pathway that commonly results in the abandonment of the technology, in the best scenario, after clinical trials (Jager et al., 2010).

Cell source in bone tissue engineering is a matter of debate, where the type of stem cell chosen for *in vitro* and *in vivo* experimentation notably differs in the literature (Gao et al., 2017). Bone marrow stromal cells (BMSCs), however, have been the preferred choice in clinical studies due to their intimate involvement in bone physiology and pathology, osteogenic potency, and anti-inflammatory properties (Zheng et al., 2019). Cell therapy for bone regeneration using freshly extracted BMSCs is a technique with 30 years of history. The first reported clinical study using bone marrow aspirates dates from 1991 (Connolly et al., 1991). In 2003, composite grafts of DBM serving as scaffolds and autologous bone marrow showed similar results as compared to autografts in the spinal fusion of 77 patients (Price et al., 2003). The same type of scaffold-cell graft showed positive results when implanted in the unicameral bone cysts of 23 patients (Rougraff and Kling, 2002). Further work showed that a collagen/hydroxyapatite composite (Healos^®^, DePuy) incubated for 20 min with autologous bone marrow aspirate promoted similar posterolateral spine fusion rates as bone autografts, avoiding any autograft-related donor-site morbidity (Neen et al., 2006). Similarly, the use of tricalcium phosphate scaffolds pre-incubated for 2 h with bone marrow aspirate, showed positive results in the spine fusion of 41 patients, 34.5 months after the procedure (Gan et al., 2008).

Despite the issues related to the *ex vivo* expansion of autologous stem cells prior to implantation, the use of this technique might also entail considerable benefits. For instance, cell expansion substantially increments cell numbers and allows for the *ex vivo* treatment of cells with growth factors or other biochemical/biophysical stimuli to increment their therapeutic potential (Cigognini et al., 2013). In a recent study, *ex vivo* expanded autologous adipose-derived stem cells (ADSCs) seeded on bioactive glass or β-tricalcium phosphate scaffolds and, in some cases, pre-incubated with BMP-2, showed integration of the constructs and tissue formation in 10 out of 13 patients suffering from large cranio-maxillofacial hard-tissue defects (Sandor et al., 2014). Furthermore, a clinical study performed in 2017 utilized a cocktail of expanded autologous BMSCs, periosteal progenitor cells and endothelial progenitor cells on a fibrin hydrogel-DBM composite, to restore critical-sized bone defects of 47 casualties with complicated gunshot bone wound. X-ray examination determined that within 4–6 months post-operatory, 90.4% of the treated defects regained native integrity (Vasyliev et al., 2017).

Taken together, tissue engineering approaches and, more precisely, the use of stem cells in combination with biomaterials has proven, in most of the clinical studies, to match or surpass the clinical outcomes of autografts. The extra step of *in vitro* cell expansion entails numerous risks and cost-related burdens but, if well designed, can increment the therapeutic outcomes. The major impediment of cell-based technologies for their clinical translation is and will be the costs and risks associated, making the address of these issues, at an early stage of research, fundamental.

## Conclusion

Despite the huge scientific efforts to develop safe and functional bone substitutes, bone tissue grafts remain the gold standard in clinical practice. The prevalence and market size of bone repair and regeneration encourage the development of therapeutic technologies to overcome limitations of bone tissue grafts and fill clinical gaps in a wide spectrum of applications (e.g., from traumatology to dentistry). Yet again, only few products have demonstrated safety and efficacy in clinical setting. Their success has been largely attributed to the precise understanding of the mechanism of action of the various device components and their compliance with regulatory frameworks. In fact, this is key in translating new concepts from lab-bench to bedside, overcoming regulatory hurdles, and normative framework changes, whilst providing a safe and functional therapy.

## Author Contributions

All authors listed have made a substantial, direct and intellectual contribution to the work, and approved it for publication.

## Conflict of Interest

PP is co-founder of GPBio Ltd. IB is a CEO and co-founder of Histograft, LLC. RD is co-founder of Histograft, LLC. DZ and RV are employees at Ilaya Medical Company. GPert and GPera are founders and shareholders of Industrie Biomediche Insubri SA. PG is President and CEO of Viscus Biologics LLC, Executive Director of Proxy Biomedical, and Director of Aran Biomedical. JB is Vice President of R&D of Viscus Biologics LLC. YB is an employee of Sofradim Production, A Medtronic Company. The remaining authors declare that the research was conducted in the absence of any commercial or financial relationships that could be construed as a potential conflict of interest.
